# Detailed statistical analysis plan for ALBINO: effect of Allopurinol in addition to hypothermia for hypoxic-ischemic Brain Injury on Neurocognitive Outcome — a blinded randomized placebo-controlled parallel group multicenter trial for superiority (phase III)

**DOI:** 10.1186/s13063-023-07828-6

**Published:** 2024-01-24

**Authors:** Corinna Engel, Mario Rüdiger, Manon J. N. L. Benders, Frank van Bel, Karel Allegaert, Gunnar Naulaers, Dirk Bassler, Katrin Klebermaß-Schrehof, Maximo Vento, Ana Vilan, Mari Falck, Isabella Mauro, Marjo Metsäranta, Sampsa Vanhatalo, Jan Mazela, Tuuli Metsvaht, Roselinda van der Vlught, Axel R. Franz, for the ALBINO Study Group, Christian F. Poets, Hercilia Guimarães, Tom Stiri, Luigi Cattarossi, Cees K. W. van Veldhuizen, Christian A. Maiwald, Iris Bergmann, Monika Weiss, Andreas Eichhorn, Michael Raubuch, Birgit Schuler, Bas Laméris, Thirza van Ramshorst, Tirol Kliniken, Johannes Brandner, Marie Tackoen, Ruth Reibel, Mari-Liis Ilmoja, Pille Saik, Ruth Käär, Pille Andresson, Klinikum der J. W. Goethe, Main Rolf Schloesser, Carl Gustav Carus, Stefan Winkler, Thomas Hoehn, Norbert Teig, Michael Schroth, Christoph Fusch, Ulrich H. Thome, Harald Ehrhardt, Ancona Virgilio Carnielli, Marcello Napolitano, Francesca Faldini, Bambini “V.Buzzi”, Milano Gianluca Lista, Mario Barbarini, Laura Pagani, Emmanuele Mastretta, Giovanni Vento, Monica Fumagalli, Mirjam M. van Weissenbruch, Henrica L. M. van Straaten, Kim V. Annink, Jeroen Dudink, Jan B. Derks, Inge P. de Boer, Clemens B. Meijssen, Timo R. de Haan, Linda G. van Rooij, Jacqueline L. van Hillegersberg, Minouche van Dongen, Koen P. Dijkman, Marlies A. van Houten, Sophie R. D. van der Schoor, Moritz Schneider, Eirik Nestaas, Britt Nakstad, Lukas Karpinski, Ewa Gulczynska, Claudia Ferraz, Almerinda Pereira, Rosalina Barroso, Mendes da Graça, Teresa Tomé, Filomena Pinto, Juan Martínez Rodilla, Maria Luz.Couce Pico, José Antonio Hurtado Suazo, Eva Valverde, José Ramón Fernández Lorenzo, Héctor Boix, Francisco Jimenez Parrilla, Dorotea Blanco, Begoña Loureiro, Maria Teresa Moral-Pumarega, Julia Maletzki, Claudia Knoepfli, Cornelia Hagmann, Michael Kleber, Martin Stocker, Thomas Riedel

**Affiliations:** 1 Center for Pediatric Clinical Studies (CPCS), University Hospital Tuebingen, Tuebingen, Germany; 2 Universitätsklinikum C. G. Carus - Medizinische Fakultät der TU Dresden, Dresden, Germany; 3 Universitair Medisch Centrum Utrecht, Utrecht, The Netherlands; 4 University Hospitals Leuven, Leuven, Belgium; 5 UniversitaetsSpital Zuerich, Zuerich, Switzerland; 6 Medizinische Universitaet Wien, Wien, Austria; 7 Hospital Universitario y Politécnico La Fe, Valencia, Spain; 8 Centro Hospitalar Universitário São João Porto, Porto, Portugal; 9 Oslo Universitetssykehus HF, Oslo, Norway; 10 Azienda sanitaria universitaria integrata di Udine, Udine, Italy; 11 Helsinki University Hospital (HUS), Helsinki, Finland; 12 Department of Neonatology, Poznan University of Medical Sciences, Poznan, Poland; 13 Tartu University Hospital, Tartu, Estonia; 14 ACE Pharmaceuticals BV, Zeewolde, The Netherlands; 15 University Hospital Tuebingen, Calwerstr. 7, 72076 Tuebingen, Germany

**Keywords:** Allopurinol, Neonatal oxygen deficiency, Hypothermia therapy, Childbirth outcome, Hypoxic-ischemic encephalopathy, Perinatal asphyxia, Brain injury, Cerebral palsy

## Abstract

**Background:**

Despite therapeutic hypothermia (TH) and neonatal intensive care, 45–50% of children affected by moderate-to-severe neonatal hypoxic-ischemic encephalopathy (HIE) die or suffer from long-term neurodevelopmental impairment. Additional neuroprotective therapies are sought, besides TH, to further improve the outcome of affected infants.

Allopurinol — a xanthine oxidase inhibitor — reduced the production of oxygen radicals and subsequent brain damage in pre-clinical and preliminary human studies of cerebral ischemia and reperfusion, if administered before or early after the insult.

This ALBINO trial aims to evaluate the efficacy and safety of allopurinol administered immediately after birth to (near-)term infants with early signs of HIE.

**Methods/design:**

The ALBINO trial is an investigator-initiated, randomized, placebo-controlled, double-blinded, multi-national parallel group comparison for superiority investigating the effect of allopurinol in (near-)term infants with neonatal HIE.

Primary endpoint is long-term outcome determined as survival with neurodevelopmental impairment versus death versus non-impaired survival at 2 years.

**Results:**

The primary analysis with three mutually exclusive responses (healthy, death, composite outcome for impairment) will be on the intention-to-treat (ITT) population by a generalized logits model according to Bishop, Fienberg, Holland (Bishop YF, Discrete Multivariate Analysis: Therory and Practice, 1975) and .”will be stratified for the two treatment groups.

**Discussion:**

The statistical analysis for the ALBINO study was defined in detail in the study protocol and implemented in this statistical analysis plan published prior to any data analysis. This is in accordance with the Declaration of Helsinki and the International Conference on Harmonization Good Clinical Practice guidelines.

**Trial registration:**

ClinicalTrials.gov NCT03162653. Registered on 22 May 2017.

**Supplementary Information:**

The online version contains supplementary material available at 10.1186/s13063-023-07828-6.

## Introduction

During labor and childbirth various events (such as placental abruption, uterine rupture, umbilical cord complications, etc.) may result in impaired oxygenation and/or perfusion of the newborn brain which may result in brain injury termed “hypoxic-ischemic encephalopathy” (HIE) (reviewed in [14]). HIE is associated with long-term motor, cognitive, and neurosensory disability, seizure disorders, and death and is one of the fundamental problems in perinatal medicine affecting about 5000–20,000 infants/year in Europe (or 1–4/1000 live births in Western societies) and approximately 1 million infants/year worldwide.

In recent years, therapeutic hypothermia became the only established therapy to improve outcomes after perinatal HIE. Despite hypothermia and modern supportive neonatal intensive care, 45–50% of children with moderate or severe HIE (i.e., 2500–10,000 infants per year in Europe) still die or suffer from long-term neurodevelopmental impairments [4]. Therefore, additional neuroprotective interventions, besides hypothermia, are warranted to further improve outcomes.

Allopurinol is a xanthine oxidase inhibitor and reduces the degradation of purines (especially adenosine) and the production of oxygen radicals and, subsequently, reduced brain damage in experimental and early human studies of ischemia and reperfusion.

This paper describes the statistical analysis plan to evaluate the efficacy and safety of allopurinol administered immediately after birth to near-term infants with perinatal asphyxia and early potential signs of HIE to attenuate long-term neurodevelopmental impairment.

The study protocol version 5 of the ALBINO study was published previously [7]. The statistical analysis was predefined in detail in the study protocol. Substantial changes were made concerning the analysis of the primary endpoint in version 6 and the definition of an interim analysis in version 7 of the study protocol. Any deviation from the originally planned statistical analysis was described within protocol amendments and accepted by ethics committees and national regulatory authorities. The statistical analysis plan (SAP) conforms with the guidelines for the content of statistical analysis plans in clinical trials [5]], please refer to checklist in supplementary material. This SAP includes the interim and the final analysis and describes the analysis principles, definition of outcomes, and methods for their analyses.

## Background information

### Rationale

Perinatal hypoxic/ischemic events can cause immediate (necrosis) and delayed death (apoptosis) of (especially neuronal) cells, the latter responsible for a substantial amount of HIE-associated permanent brain damage. Whereas no intervention is known to prevent necrosis, the delayed cell death by apoptosis can be reduced by therapeutic interventions:

Apoptosis is in part caused by secondary energy failure which can be reduced by hypothermic treatment [6, 4, 13].

Apoptosis is also caused by xanthine oxidase-mediated production of cytotoxic oxygen radicals during reperfusion, and there is evidence that allopurinol, a xanthine-oxidase inhibitor, reduces delayed cell death in animal models of perinatal asphyxia and ischemia/reperfusion [9, 15, 3]. Allopurinol, a xanthine-oxidase inhibitor, blocks purine degradation. It also seems to result in the accumulation of adenosine during hypoxia, since allopurinol treatment increases brain tissue levels of adenosine after hypoxic-ischemic injury [8]. Adenosine is a potent inhibitory neuromodulator providing additional neuroprotection in HIE. In higher concentrations, allopurinol acts as an iron chelator and direct scavenger of free radicals [12]. Allopurinol pretreatment preserves cerebral energy metabolism as shown by 31P NMR during perinatal hypoxia-ischemia in immature rats [17], and thus prevents cerebral damage [9].

The evidence for a potential neuroprotective effect of allopurinol and the preclinical and early clinical studies on allopurinol for HIE have been reviewed [1]. The suggested neuroprotective effect is the basis of the ALBINO study, which has been described in detail in the publication of the study protocol [7].

### Objectives

The primary objective of the ALBINO trial is to determine whether in newborns with perinatal asphyxia and early clinical signs of HIE, early postnatal allopurinol compared to placebo administered in addition to standard of care (including therapeutic hypothermia if indicated) reduces the incidence of death or severe neurodevelopmental impairment (defined as cerebral palsy, or cognitive or language impairment) at 24 months of age.

The secondary objectives are to evaluate the effect of allopurinol in addition to hypothermia (if indicated) on biomarkers such as:

- Brain injury assessed by magnetic resonance imaging
- Brain injury assessed by (amplitude integrated) electroencephalogram

### Trial design

This is an investigator-initiated, randomized, placebo-controlled, (double-)blinded, multi-national, parallel-group comparison for superiority (phase III study) of allopurinol compared to placebo in preventing death or neurodevelopmental impairment at 24 months postnatal age in infants with perinatal asphyxia and early signs of evolving HIE. Essential components of its study protocol have been published [7] and, subsequently, a protocol amendment introducing an interim analysis after 300 included infants reached 24 months postnatal age was approved by ethics committees and authorities in 2023.

### Eligibility

#### Inclusion criteria

Term and near-term infants with a history of disturbed labor who meet at least one criterion of severe perinatal acidosis (or ongoing resuscitation) (as a surrogate for “asphyxia”) are considered eligible for this study.

Criteria for severe perinatal acidosis are defined as

- Umbilical (or arterial or reliable venous) blood gas within 30 min after birth with pH < 7.0
- Umbilical (or arterial or reliable venous) blood gas within 30 min after birth with base deficit ≥ 16 mmol/l (i.e., a base excess ≤ − 16 mmol/l)
- Need for an ongoing cardiac massage at/beyond 5 min postnatally
- Need for adrenalin administration during resuscitation
- APGAR score ≤ 5 at 10 min

Additionally, at least two out of the following four criteria of evolving HIE must be met:

- Altered state of consciousness (reduced or absent response to stimulation or hyperexcitability)
- Severe muscular hypotonia or hypertonia,
- Absent or insufficient spontaneous respiration (e.g., gasping only) with the need for respiratory support at 10 min postnatally
- Abnormal primitive reflexes (absent suck or gag or corneal or Moro reflex) or abnormal movements (e.g., potential clinical correlates of seizure activity)

#### Exclusion criteria

- Gestational age below 36 weeks
- Birth weight below 2500 g
- Postnatal age >30 min at the end of the screening phase
- Severe congenital malformation or syndrome requiring neonatal surgery or affecting long-term outcome
- Patient considered “moribund”/“non-viable” (e.g., lack of spontaneous cardiac activity and ongoing chest compression at 30 min)
- Decision for “comfort care only” before study drug administration
- Parents declined study participation as a response to measures of community engagement
- Both parents are insufficiently fluent in the study site’s national language(s) or English or do not seem to have the intellectual capacity to understand the study procedures and to give consent as judged by the personnel who had been in contact with the mother/father before delivery.
- Both parents/guardians less than 18 years of age, in case of single parent/guardian this one less than 18 years of age

### Interventions

Allopurinol, as a powder for injection (PFI), is administered in two doses. The first dose (20 mg/kg in 2 ml/kg sterile water for injection) is given as soon as intravenous access is established. The start of infusion of study medication should be within 30 min (no later than 45 min) after birth and the second dose (10mg/kg in 1ml/kg sterile water for injection) 12 ± 0.5 h after the (beginning of the infusion of the) first dose. The second dose will only be administered to infants treated with therapeutic hypothermia. Infants who recover quickly and do not qualify for and hence do not undergo hypothermia do not receive a second dose. Administration is by infusion over 10 min using a syringe pump through secure venous access.

Mannitol infusion, as a powder for injection (PFI), is given as a placebo treatment. Dosing and application are the same as for allopurinol, that is 20 mg/kg in 2 ml/kg sterile water given as soon as intravenous access is established (within 45 min after birth) followed by a second dose (10 mg/kg in 1 ml/kg sterile water) — administered over 10 min using a syringe pump through secure venous access, where the second dose is only given in infants that were treated with therapeutic hypothermia.

### Definition of primary and secondary outcomes

#### Primary outcome

The primary endpoint is defined as three mutually exclusive outcomes: survival with neurodevelopmental impairment (NDI, defined as cerebral palsy or severe cognitive and/or language delay at 2 years) or death or survival without NDI at 2 years postnatal age.

#### Secondary outcomes

- Death or survival with NDI versus survival without NDI (primary endpoint will be reconstituted as dichotomized composite secondary outcome — survival without NDI versus Death or NDI)
- Posterior probability that treatment is better than placebo concerning the rate of healthy survivors (survival without NDI), estimated using a Bayesian approach.
- Incidence of Death
- Incidence of cerebral palsy (CP)
- Gross motor function classification system (GMFCS)-Score
- Motor-Composite-Score of the Bayley III
- Cognitive-Composite Score of the Bayley III
- Language-Composite Score of the Bayley III

#### Further relevant endpoints

- Anthropometric measures, neurological status, milestones, seizure activity, as well as visual and hearing impairment at 2-year follow-up (in detail: cognitive and language score of PARCA-R-questionnaire, progress concerning weight, head circumference and length, incidence of severe visual and hearing impairment, neurological status, milestones at follow-up concerning right and left hand as well as leg control and speech, incidence of persisting seizure activity and need for anticonvulsive therapy)
- Results of central reading of magnetic resonance Imaging (MRI) (in detail: Weeke scores [16] and ADC-map-measurement)
- Results of central reading of electroencephalogram (EEG) epochs 0–12, 12–24 h, 24–48 h, 48–72 h, and 72–96 (in detail: most abnormal background pattern, dominant background pattern, seizure activity, time from birth until onset of any appreciable sleep-wake cycling, time from birth until onset of fully developed sleep-wake cycling, time from birth until onset of first normalization of aEEG trace)

### Level of significance

The intention-to-treat (ITT) population is the basis for the confirmatory analysis of the primary endpoint with a significance level of 0.001 in the interim analysis and 0.05 in the final analysis (according to Peto/Haybittle [10]). The analysis of the primary endpoint is based on the PP population and all analyses of secondary and further endpoints are descriptive and will be regarded as remarkable if *p* < 0.05.

### Sample size and power

An incidence of death or severe NDI of 27% in the Allopurinol group compared to 37% in the control group is expected. A total of 682 infants (341 per treatment group) will be required in whom the primary outcome can be ascertained. Assuming a drop-out rate of 10% for loss to follow-up, a total of 760 infants need to be enrolled with formal written consent. Assuming that 10% of parents will refuse continuing participation after the initial dose of the study drug (following short oral or deferred consent procedures, depending on the country) 846 infants have to be randomized immediately after birth.

Intervention allocation and blinding

Clinicians, caregivers, and trial outcome assessors are masked.

Randomization lists have been prepared by the CPCS and were sent to ACE Pharmaceuticals for blinded labeling and packaging of the study medication. Randomization has been done in blocks of four in a 1:1 ratio, with an equal number of patients in each treatment arm. Each shipment of study medication to study centers comprised complete blocks of 4, thereby achieving stratification by center and allocation concealment.

Justification:

Although a variable block size would have been desirable for best allocation concealment, a fixed block size of 4 was selected for the prevention of an uneven distribution of verum/placebo in this study with a low anticipated recruitment rate per center (on average < 10–15) — as well as for practical reasons of study medication distribution to numerous study sites.

Stratification for therapeutic hypothermia — although desirable — was impossible, because the clinical indications for therapeutic hypothermia evolve with time and may not be apparent at the 1st dose of study medication.

#### Data collection schedule

Data are collected in electronic case record forms (eCRFs) into the study’s secuTrial® electronic database by the staff of each participating center.

The eCRFs to be completed are as follows:

- Screening
- Randomization and first dose of study medication
- Full written informed consent
- Baseline — infant data
- Baseline — maternal data
- Hypothermia treatment and 2nd dose of study medication
- Thompson score at 1–6 h and 84–106 h or discharge (whichever comes first)
- Blood gas analysis
- Cell injury markers and documentation of hypereosinophilia
- Medication before or during aEEG and mchEEG measurement
- Neonatal outcome until day 14
- Discharge
- Follow-up (Overall, Bayley, Parent Questionnaires^1^)
- End of study
- Adverse events

The following documentation will be done in the respective eCRFs after central assessment by the respective staff (Table 1):

- MRI central reading
- aEEG central reading
- Cerebral ultrasound (0–24 h, 48–72 h, 96–120 h)
- Peroxidase products (blood)
- Peroxidase products (urine)
- S100B and inflammasome-mediated cytokines

**Table 1:** Study examinations and data collection

***Procedure***	***Initial hospitalization***			***Outpatient***
***Time range from birth***	**0–24 h**	**At a predefined time before discharge**	**discharge**	**2 years**
***Screening***	✓			
***Randomization and first dose of study medication***	✓			
***Full written informed consent***		✓		
***Baseline — infant data***		✓		
***Baseline — maternal data***		✓		
***Hypothermia treatment and 2nd dose of study medication***		✓		
***Thompson score at 1–6 h and 84–106 h or discharge (whichever comes first)***	✓	✓		
***Blood gas analysis***	✓✓			
***Cell injury markers and hypereosinophilia***	**√**	✓		
***Neonatal outcome until day 14***		✓		
***aEEG (in case of hypothermia until 84 h)***	✓			
***mchEEG***		✓		
***Peroxidase products, blood***	✓✓			
***Peroxidase products, urine***	✓✓			
***Head ultrasound***	✓	✓✓		
***S100B and inflammasome-mediated cytokines***	✓	✓		
***MRI***		✓		
***Discharge***			✓	
***Adverse events***	**Continuously**			
***Follow-up***				✓
***End of study***				✓

#### Regular safety reporting to the DMC

Safety reporting to the data monitoring committee (DMC) is done after 10, 30, 50, 100, 200, 300, 400, and 600 patients have reached 44 weeks postmenstrual age (PMA).

The following safety parameters are reported and were predefined in the study protocol and described in detail in the DMC charter:

- Patient characteristics (birth weight, gestational age at birth, gender, umbilical artery pH and lactate)
- Compliance with the protocol (1st dose of study medication (age at start of administration and administered dose), 2nd dose of study medication (administered dose and interval after 1st dose)
- Safety parameters (mortality, HIE severity, blood gas analyses at 0.5–6 h/6 to 12 h/12 to 24 h after birth, cell injury markers and plasma osmolality at 24 h ± 6 h after birth, results of portal vein ultrasound in the subgroup of patients with administration of medication through umbilical venous catheter, potential clinical/laboratory signs of allopurinol hypersensitivity reactions, organ failure until day 14 after birth or discharge home (whichever comes first), clinical seizures, health status, support on discharge, MRI — Weeke scores)
- Listings and aggregate summary tabulation of adverse reactions and adverse events

#### Interim analysis and stopping rules

No interim analysis was intended at the beginning of the study due to the fact that the primary endpoint will be determined at 2 years follow-up, and recruitment should have been already terminated before 50% of recruited infants have reached 2 years of age according to the original recruitment plan.

A protocol amendment (protocol version 7) approved in 2023 defined an interim analysis after 300 patients had reached follow-up within 2 years. Interim Analysis will be done according to Peto/Haybittle [10]) on a two-sided level of significance of 0.001, leaving a two-sided level of significance of 0.05 for the final analysis.

Depending on the result of the interim analysis, the study will be stopped or continued according to predefined criteria:

1. In the event that the null hypothesis of equal proportions of primary endpoint in the two groups is rejected (based on a two-sided *p*-value <0.001), the trial statistician will recommend immediate stop of recruitment.
2. Also, in the event that the point estimate for the rate of the primary outcome “survival without severe neurodevelopmental impairment” is exactly equal for the experimental group compared to the placebo group, the trial statistician will recommend an immediate stop of recruitment for the futility of the trial.
3. In all other cases, the results will be reported directly to the members of the independent DMC with data being identified as Group A or Group B first. The DMC may request unblinding.

The DMC will decide about whether or not to advise the steering committee to discontinue the study. “*Proposed*” decisions depending on the results of the interim analysis of the primary outcome are listed in Table 2 (for guidance), but safety data will additionally be considered and biomarker data may be taken into account.

**Table 2:** Proposed decisions depending on results of the interim analysis of the primary outcome

**Result of interim analysis**	**Resulting new sample size**^**a**^	**Proposed recommendation**
Risk reduction in the experimental group much smaller than expected before the start of the study.	> 780	Immediate discontinuation of recruitment, because sample size cannot be reached with the available resources.
Risk reduction in the experimental group smaller than expected before the start of the study.	> 680–780 (resulting in a prolongation of recruitment of about one year)	Consider to extend recruitment according to the new sample size. No protocol amendment needed due to prolongation of at maximum 1 year.
Risk reduction in the experimental group as expected before the start of the study.	680	Continue to full pre-defined sample size.
Risk reduction in the experimental group higher than expected before the start of the study.	<680	Continue recruitment until actually needed (reduced) sample size is reached.

^a^No. of patients with primary endpoint ascertained to be analyzed for confirmatory analysis (power 80%)

No adjustment of the significance level due to interim analysis will be done.

In addition to the analysis of the primary endpoint, the DMC will be provided with the data usually included in DMC reports (refer to section “DMC reporting”).

If the DMC recommends to continue the study, the detailed results of the interim analysis will not be communicated to any person directly involved in the conduct of the trial until the final analysis will be done.

## Trial reporting

The trial will be reported according to the CONSORT principles [11]. The final analysis will be done after a 2-year follow-up of the last patient.

### Protocol non-compliances

Regular remote and on-site monitoring according to a predefined monitoring manual ensures high quality of the data. The following protocol non-compliances will be listed in the final report:

#### Major

- Any protocol deviation that may influence the results of the study
- This may include the following deviations from the protocol that will lead to exclusion from the per-protocol population:
- Participants randomized in error, i.e., not fulfilling all inclusion or fulfilling an exclusion criterion
- Time to administration of 1st dose exceeded 45min postnatally
- Deviation of actually administered dose by more than 10% from the intended dose
- Any open-label allopurinol

#### Minor

- Protocol deviations that will likely not influence study results (e.g., incorrect timing of a brain ultrasound/blood sample)

### Treatment non-compliances

- Timing and dose of study medication, are subject to source data verification by monitoring.
- Participants in whom the above-listed major protocol deviations occurred will be excluded from the per-protocol population

### Analysis populations

#### Post-randomization exclusions

No post-randomization exclusions will be done except for the case that there would be a patient for whom fraudulent data are detected.

#### Population definitions

##### Intention-to-treat population

The intention-to-treat population (ITT) will be all patients included in the study. Patients for whom informed consent was withdrawn will be included in the analysis with all their data that were collected before the withdrawal of consent.

##### Interim analysis population

The interim analysis will include all randomized patients with the date of birth before the pre-defined reference date minus 24 months.

##### Safety population

The safety population consists of all patients included in the study.

## Descriptive analyses

Numerical items will be summarized as number, number missing, mean, standard deviation, minimum, q25, median, q75, and maximum, if appropriate. Categorical items will be summarized as numbers and percentages.

## Representativeness of the trial population and participants throughout

Participant’s flow through each stage of the study will be presented in a CONSORT scheme.

## Baseline characteristics of treatment groups

Baseline characteristics of infants and their mothers will be described for the ITT population stratified for the treatment group. The characteristics presented are:

### Mother’s baseline characteristics

- Age in years
- Maternal ethnic background
- Pregnancy-related items (diabetic condition, hypertensive disorder, pathological umbilical or Doppler examination)
- Delivery-related items (mode of delivery, general anesthesia before delivery, clinical chorioamnionitis, uterine rupture, placental abruption, cord complications, other complications

### Infant’s characteristics at trial entry

- Demographic data (gender, place of birth)
- Basic infant data (birthweight, head circumference, length at birth, APGAR score at 5 and 10 min, late umbilical cord clamping or milking of the cord)
- Blood gas analysis (pH, lactate, base excess)
- Delivery room and NICU data (body temperature on admission to NICU/neonatal ward, duration of bag and mask ventilation — if done, age at ET-tube placement — if done, age at the resumption of spontaneous respiration if resumed in the delivery room, age at return of sufficient spontaneous circulation if in the delivery room, cumulative dose of crystalloid volume, colloid volume, erythrocyte concentrate, adrenalin, and bicarbonate — if any, suspected meconium or blood aspiration, suspected sepsis)

## Losses to follow-up

**Fig. 1:**
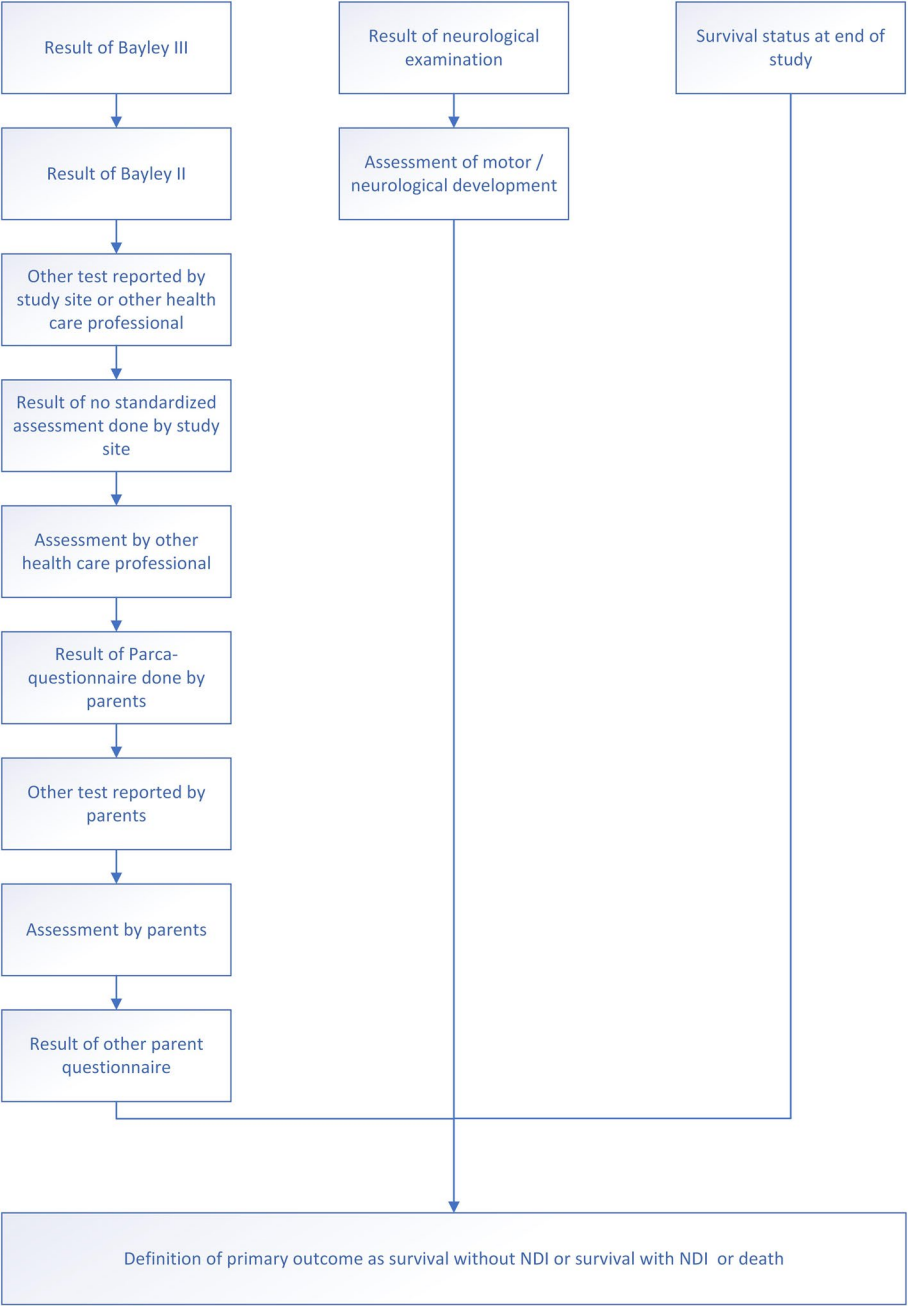
Definition of primary endpoint. If data are not available for the first level of definition, the second level applies, and so on. NDI, neurodevelopmental impairment

The primary endpoint will be assessed at follow-up within 2 years. We expect a certain proportion of patients that will withdraw their informed consent or be lost to follow-up. To avoid losses to follow-up several other sources for information on neurodevelopmental outcomes besides regular follow-up at the study center and the performance of the Bayley III examination will be taken into account (refer to the “Primary endpoint” section and Fig. 1).

## Comparative analyses

According to the intention-to-treat principle infants will be analyzed according to the treatment group they were randomized to, regardless of the treatment they may have received.

Numerical items will be summarized as number, number missing, mean, standard deviation, minimum, 25% quantile, median, 75% quantile, and maximum, if appropriate. Categorical items will be summarized as numbers and percentages.

Comparative analyses will be stratified for the treatment group only. Due to the fact that there are so many centers participating in this study, the analyses will not be stratified for centers. This is in accordance with ICH E9 for multicenter trials if it is recognized from the start that the limited numbers of subjects per center will make it impracticable to include the center effects in the statistical models.

## Detailed definition of outcomes

The primary outcome is composed by cognitive and language development, motor development, and survival status at the age of 2 years.

The assessment of motor development is based on the presence or absence of cerebral palsy (CP). CP is diagnosed if the child has a non-progressive motor impairment characterized by abnormal muscle tone and impaired range or control of movements, according to the criteria defined by the European network “Surveillance of CP in Europe.” A severely abnormal neurological status is classified as unilateral spastic CP, bilateral spastic CP, ataxic CP, dyskinetic CP, or no CP, but other severe abnormality.

To avoid missing data the following hierarchy applies:

- Neurological classification by the study team indicates cerebral palsy
- Assessment of motor/neurological development by other health care professionals or other sources (i.e., parents) indicates cerebral palsy

Cognitive and language development is based on the presence or absence of an abnormal cognitive and/or language development assessed by the cognitive-composite-score and the language-composite-score on the Bayley Scales of Infant and Toddler Development (3rd edition). An abnormal development is defined as a composite score of < 85 in at least one of the two scales.

To avoid missing data the following hierarchy applies:

- Bayley III: Composite cognition score and/or language cognition score < 85
- Explanation why composite cognition and/or language score has not been provided despite Bayley III having been attempted is indicating abnormal cognitive and/or abnormal language development
- Bayley II: mental development index (MDI) < 85
- Rating done by health care professional: other test, assessment, or reason for no test indicates abnormal cognitive and/or abnormal language development
- PARCA-R (documented by parents) with language and/or cognition standardized score <85
- Rating done by other source, i.e., parents: other test, assessment, or reason for no test indicates abnormal cognitive and/or abnormal language development
- Other parental questionnaire results indicate abnormal neurodevelopment

The hierarchy of the definition of the primary endpoint is displayed in Fig. 1.

Detailed derivations of all other outcomes are described in a separate document.

### Primary analysis

Primary endpoint with three mutually exclusive responses (healthy, death, composite outcome for impairment) will be analyzed — stratified for the two treatment groups — by a generalized logits model according to Bishop, Fienberg, Holland [2] with SAS 9.4 procedure proc catmod within the intention-to-treat (ITT) population.

### Secondary analysis

Secondary endpoints will be analyzed by the Cochrane-Mantel-Haenzel-*χ*^2^-test in case of categorial binary data and by the Wilcoxon-Mann-Whitney test in case of score data. Bayley-III-scores will be cut due to lack of sensitivity below 50 points and therefore only fit for non-parametric methods.

Bayesian approach will be done by SAS 9.4 procedure proc genmod.

### Analysis of further relevant endpoints

Further relevant endpoints will be analyzed by the Cochrane-Mantel-Haenzel-*χ*^*2*^-test in the case of categorial binary data. Numerical endpoints will be analyzed using parametric or non-parametric methods as appropriate. The proportional odds model will be applied for the analysis of non-binary categorial endpoints.

### Multivariate analyses

Multivariate analyses of the primary endpoint will be done including gender, postnatal age at administration of a first dose of study medication (< 15 min after birth vs. 16–30 min after birth vs. >30 min after birth), encephalopathy, where the degree of HIE severity will be derived from the Thompson Score assessed at 3–6 h (before hypothermia) and the initial aEEG findings (first epoch and before any brain-acting medication and/or hypothermia), need for therapeutic hypothermia (yes versus no).

Analysis will be performed by a generalized logits model according to Bishop, Fienberg, Holland [2] with SAS 9.4 procedure proc catmod, adjusted for the treatment group.

The final multivariate model will only include those risk factors with *p*-value <0.05. These will be checked for interactions one interaction term at a time. A term will suggest an interaction if it reveals a *p*-value <0.05.

Appropriate subgroup analyses will be performed if these multivariate analyses suggest an interaction between the intervention and one of the risk factors. These post-hoc subgroup analyses are meant to be exploratory (hypotheses generating).

## Significance levels and adjustment of p-values for multiplicity

All analyses will be done to assess the superiority of the study medication compared to placebo treatment. Only the result of the analysis of the primary endpoint in the intention-to-treat population will be regarded to be confirmative. Consequently, no adjustment of *p*-values for multiplicity will be done.

### Missing data and sensitivity analysis

In case of more than 10% missing values after hierarchical substitution of data concerning the primary outcome as described in the “Detailed definition of outcomes” section and Fig. 1, a worst case/best case analysis for this endpoint will be performed in the intention-to-treat population as sensitivity analyses and results will be included in the final report.

No imputation will be done for secondary or further relevant endpoints.

## Statistical software employed

SAS 9.4 will be used for all analyses.

## Additional exploratory analysis

Analyses not specified in the study protocol and the statistical analysis plan will be exploratory in nature and have to be defined in a separate statistical analysis plan. Any post hoc analysis requested by the DMC, a journal editor, or anyone else will be labeled explicitly as such.

## Discussion

This article presents the statistical analysis plan for the ALBINO study which has been described in detail in the study protocol and has been substantially changed twice by choosing a more powerful analysis strategy for the primary outcome and by defining an interim analysis. Both substantial changes were included in protocol amendments, approved by ethics committees and national authorities, and implemented before any analysis was started.

### Strengths

This is a state-of-the-art randomized trial in a challenging indication and study population. Even if closed after interim analysis, the Albino study will be one of the largest studies so far to evaluate the efficacy and safety of pharmaceutical intervention to improve long-term outcomes after perinatal asphyxia in the era of therapeutic hypothermia and the only one that faced the challenge of administration of study medication immediately after birth. This study will give valuable insight into the application of biomarkers for HIE and the effect of allopurinol.

### Limitations

The limitations of the study are the struggle with several problems concerning approval by national authorities and the down-scaling of allopurinol to achieve a pediatric formulation. This caused a delayed start of the recruitment. Additionally, recruitment has been very slow due to a limited number of suitable inborn patients at the participating almost 70 trial sites and the fact that in the majority of sites short oral consent by at least one parent has to be achieved within the first 45 min after birth. Due to limited resources, this may necessitate preterm termination of the study before reaching the calculated sample size potentially preventing conclusive results.

## Trial status

At present, the study has been registered at www.clinical.trials.gov (NCT03162653, on May 22, 2017) and the study protocol has been published [7]. The first patient in was on March 27, 2018, and the status of recruitment is at 460 patients recruited and 300 recruited patients have reached 2 years’ postnatal age.

## Deviation from analysis described in protocol

None yet.

### Supplementary Information

**Additional file 1.**

## Data Availability

Data sharing is not applicable to this article as no datasets were generated or analyzed during the current study.

## Abbreviations

CFR: Code of Federal Regulations
CP: Cerebral palsy
CPCS: Center for Pediatric Clinical Studies
DMC: Data monitoring committee
eCRF: Electronic case report form
EEG: Electroencephalogram
FDA: Food and Drug Administration
GCP: Good clinical practice
GMFCS: Gross motor function classification system
HIE: Hypoxic-ischemic encephalopathy
ICH: International Conference of Harmonization
ITT: Intention-to-treat
MDI: Mental development index
MRI: Magnetic resonance imaging
NDI: Neurodevelopmental impairment
PFI: Powder for injection
PMA: Postmenstrual age
PP: Per protocol
SAP: Statistical analysis plan
TH: Therapeutic hypothermia

## Footnotes

1 Paper version of PARCA-R questionnaire will be filled by parents and inserted into the eCRF by the staff of each center.
