# Correction: Detailed statistical analysis plan for ALBINO: effect of Allopurinol in addition to hypothermia for hypoxic-ischemic Brain Injury on Neurocognitive Outcome — a blinded randomized placebo-controlled parallel group multicenter trial for superiority (phase III)

**DOI:** 10.1186/s13063-024-08031-x

**Published:** 2024-03-15

**Authors:** Corinna Engel, Mario Rüdiger, Manon J. N. L. Benders, Frank van Bel, Karel Allegaert, Gunnar Naulaers, Dirk Bassler, Katrin Klebermaß-Schrehof, Maximo Vento, Ana Vilan, Mari Falck, Isabella Mauro, Marjo Metsäranta, Sampsa Vanhatalo, Jan Mazela, Tuuli Metsvaht, Roselinda van der Vlught, Axel R. Franz, for the ALBINO Study Group

**Affiliations:** 1 Center for Pediatric Clinical Studies (CPCS), University Hospital Tuebingen, Tuebingen, Germany; 2 Universitätsklinikum C. G. Carus - Medizinische Fakultät der TU Dresden, Dresden, Germany; 3 Universitair Medisch Centrum Utrecht, Utrecht, The Netherlands; 4 University Hospitals Leuven, Leuven, Belgium; 5 UniversitaetsSpital Zuerich, Zuerich, Switzerland; 6 Medizinische Universitaet Wien, Wien, Austria; 7 Hospital Universitario y Politécnico La Fe, Valencia, Spain; 8 Centro Hospitalar Universitário São João Porto, Porto, Portugal; 9 Oslo Uni- versitetssykehus HF, Oslo, Norway; 10 Azienda sanitaria universitaria integrata di Udine, Udine, Italy; 11 Helsinki University Hospital (HUS), Helsinki, Finland; 12 Department of Neonatology, Poznan University of Medical Sciences, Poznan, Poland; 13 Tartu University Hospital, Tartu, Estonia; 14 ACE Pharmaceuticals BV, Zeewolde, The Netherlands; 15 University Hospital Tuebingen, Calwerstr. 7, 72076 Tuebingen, Germany

**Correction: Trials 25, 81 (2024)**

**https://doi.org/10.1186/s13063-023-07828-6**

Following publication of the original article [1], we have been notified that updates were needed to the Recruiting Hospitals and Local Principal Investigator list of the ALBINO study group in the acknowledgement section.

Names originally included, but removed with this correction:

Germany: **Stefan Winkler**, Universitätsklinikum C. G. Carus - Medizinische Fakultät der TU Dresden, **Barbara Seipold**, Universitätsklinikum C. G. Carus - Medizinische Fakultät der TU Dresden

Names originally missing, but included with this correction:

Austria: **Martin Wald**

Finland: **Kristiina Saarinen** and **Krista Rantakari**

Germany: **Lübeck Philipp Jung**, Universitätsklinik für Kinder- und Jugendmedizin, **Birgit Kampschulte**, Department of General Pediatrics and Neonatology, Justus‑Liebig‑University Gießen, **Thomas Michael Volkl**, KJF Klinik Josefinum, **Anna Koluch**, AUF DER BULT Kinder- und Jugendkrankenhaus Hannover, **Patrick Mohrhart**, Universitätsklinikum Erlangen, Jenny Potratz, Universitätsklinikum Münster Torsten Ott

Netherlands: **Gerbrich Engelien van den Bosch**, Erasmus MC – Sophia Children’s Hospital, **Marianne van der Heide**, Isala Kliniek Zwolle, **Carien C.C.J.M Smeets**, Sint Elisabeth Ziekenhuis Tillburg

Spain: **Ana Concheiro Guisá**, Complejo Hospitalario Universitario Vigo

Switzerland: **Mara Hesse**, UniversitaetsSpital Zuerich, **Bjarte Rogdo**, Kantonsspital Graubünden (Chur), **Philipp Meyer**, Kantonsspital Aarau AG, **Vera Bernet**, Spital Zöllikerberg

The original article has been corrected.
