# Supplementary material for: Detailed statistical analysis plan for ALBINO: effect of Allopurinol in addition to hypothermia for hypoxic-ischemic Brain Injury on Neurocognitive Outcome — a blinded randomized placebo-controlled parallel group multicenter trial for superiority (phase III)
Source: Trials. 2024 Jan 24;25:81. doi: 10.1186/s13063-023-07828-6 (PMC10809613; doi:10.1186/s13063-023-07828-6)
Supplement: Supplementary file 1 — Additional file 1. [file 13063_2023_7828_MOESM1_ESM.docx]

**Checklist for statistical analysis plan concerning accordance with “Guidelines for the Content of Statistical Analysis Plans in Clinical Trials”**

(JAMA 2017 (318(23):2337-2343. doi:10.1001/jama.2017.18556)

Name of the study: Albino

Date: June 2023

Author of SAP: Dr. Corinna Engel

| **Section / Item** | **Index** | **Description** | **Included in SAP** |
| --- | --- | --- | --- |
| **Section 1: Administrative Information** |  |  |  |
| Title and trial registration | 1a | Descriptive title that matches the protocol, with SAP either as a forerunner or subtitle, and trial acronym (if applicable) | X |
|  | 1b | Trial registration number | X |
| SAP version | 2 | SAP version number with dates | X |
| Protocol version | 3 | Reference to version of protocol being used | X |
| SAP versions | 4a | SAP revision history | X |
|  | 4b | Justification for each SAP version | X |
|  | 4c | Timing of SAP revisions in relation to interim analysis, etc. | X |
| Roles and responsibilities | 5 | Names, affiliations, and roles of SAP contributors | X |
| Signatures of: | 6a | Person writing the SAP | X |
|  | 6b | Senior statistician responsible | (X) |
|  | 6c | Chief investigator / clinical head | X |
| **Section 2: Introduction** |  |  |  |
| Background and rationale | 7 | Synopsis of trial background and rationale including a brief description of research question and brief justification for undertaking the trial | X |
| Objectives | 8 | Description of specific objectives or hypotheses | X |
| **Section 3: Study Methods** |  |  |  |
| Trial design | 9 | Brief description of trial design including type of trial (eg, parallel group, multiarm, crossover, factorial) and allocation ratio and may include brief description of interventions | X |
| Randomization | 10 | Randomization details, eg, whether any minimization or stratification occurred (including stratifying factors used or the location of that information if it is not held within the SAP) | X |
| Sample size | 11 | Full sample size calculation or reference to sample size calculation in protocol  (instead of replication in SAP) | X |
| Framework | 12 | Superiority, equivalence, or noninferiority hypothesis testing framework, including which comparisons will be presented on this basis | X |
| Statistical interim analyses and stopping guidance | 13a | Information on interim analyses specifying what interim analyses will be carried out  and listing of time points | X |
|  | 13b | Any planned adjustment of the significance level due to interim analysis | X |
|  | 13c | Details of guidelines for stopping the trial early | X |
| Timing of final analysis | 14 | Timing of final analysis, eg, all outcomes analyzed collectively or timing stratified  by planned length of follow-up | X |
| Timing of outcome assessments | 15 | Time points at which the outcomes are measured including visit “windows” | X |
| **Section 4: Statistical Principles** |  |  |  |
| Confidence intervals and P values | 16 | Level of statistical significance | X |
|  | 17 | Description and rationale for any adjustment for multiplicity and, if so, detailing how the type 1 error is to be controlled | X |
|  | 18 | Confidence intervals to be reported | na |
| Adherence and protocol deviations | 19a | Definition of adherence to the intervention and how this is assessed including extent of exposure | X |
|  | 19b | Description of how adherence to the intervention will be presented | X |
|  | 19c | Definition of protocol deviations for the trial | X |
|  | 19d | Description of which protocol deviations will be summarized | X |
| Analysis populations | 20 | Definition of analysis populations, eg, intention to treat, per protocol,  complete case, safety | X |
| **Section 5: Trial Population** |  |  |  |
| Screening data | 21 | Reporting of screening data (if collected) to describe representativeness  of trial sample | x |
| Eligibility | 22 | Summary of eligibility criteria | x |
| Recruitment | 23 | Information to be included in the CONSORT flow diagram | x |
| Withdrawal/follow-up | 24a | Level of withdrawal, eg, from intervention and/or from follow-up | x |
|  | 24b | Timing of withdrawal/lost to follow-up data | x |
|  | 24c | Reasons and details of how withdrawal/lost to follow-up data will be presented | x |
| Baseline patient characteristics | 25a | List of baseline characteristics to be summarized | X |
|  | 25b | Details of how baseline characteristics will be descriptively summarized | X |
| **Section 6: Analysis** |  |  |  |
| Outcome definitions |  | List and describe each primary and secondary outcome including details of: |  |
|  | 26a | specification of outcomes and timings. If applicable include the order of importance of primary or key secondary end points (eg, order in which they will be tested) | X |
|  | 26b | specific measurement and units (eg, glucose control, hbA1c [mmol/mol or %]) | X |
|  | 26c | any calculation or transformation used to derive the outcome (eg, change from baseline, QoL score, time to event, logarithm, etc) | X |
| Analysis methods | 27a | what analysis method will be used and how the treatment effects will be presented | X |
|  | 27b | any adjustment for covariates | X |
|  | 27c | methods used for assumptions to be checked for statistical methods | X |
|  | 27d | details of alternative methods to be used if distributional assumptions do not hold, eg, normality, proportional hazards, etc | X |
|  | 27e | any planned sensitivity analyses for each outcome where applicable | X |
|  | 27f | any planned subgroup analyses for each outcome including how subgroups are defined | X |
| Missing data | 28 | Reporting and assumptions/statistical methods to handle missing data (eg, multiple imputation) | X |
| Additional analysis | 29 | Details of any additional statistical analyses required, eg, complier-average causal effect^10^ analysis | na |
| Harms | 30 | Sufficient detail on summarizing safety data, eg, information on severity, expectedness, and causality; details of how adverse events are coded or categorized; how adverse event data will be analyzed, ie, grade 3/4 only, incidence case analysis, intervention emergent analysis | X |
| Statistical software | 31 | Details of statistical packages to be used to carry out analyses | X |
| References | 32a | References to be provided for nonstandard statistical methods | X |
|  | 32b | Reference to Data Management Plan | X |
|  | 32c | Reference to the Trial Master File and Statistical Master File | X |
|  | 32s | Reference to other standard operating procedures or documents to be adhered to | X |
